# Tau physiology and pathomechanisms in frontotemporal lobar degeneration

**DOI:** 10.1111/jnc.13600

**Published:** 2016-06-15

**Authors:** Liviu‐Gabriel Bodea, Anne Eckert, Lars Matthias Ittner, Olivier Piguet, Jürgen Götz

**Affiliations:** ^1^ Clem Jones Centre for Ageing Dementia ResearchQueensland Brain InstituteThe University of QueenslandBrisbaneQLDAustralia; ^2^ Neurobiology LaboratoryPsychiatric University Clinics BaselUniversity of BaselBaselSwitzerland; ^3^ Dementia Research UnitSchool of Medical SciencesFaculty of MedicineUniversity of New South WalesSydneyNSWAustralia; ^4^ Neuroscience Research AustraliaSydneyNSWAustralia

**Keywords:** microtubule, phosphorylation, post‐translational, spreading, synapse, transgenic

## Abstract

Frontotemporal lobar degeneration (FTLD) has been associated with toxic intracellular aggregates of hyperphosphorylated tau (FTLD‐tau). Moreover, genetic studies identified mutations in the *MAPT* gene encoding tau in familial cases of the disease. In this review, we cover a range of aspects of tau function, both in the healthy and diseased brain, discussing several *in vitro* and *in vivo* models. Tau structure and function in the healthy brain is presented, accentuating its distinct compartmentalization in neurons and its role in microtubule stabilization and axonal transport. Furthermore, tau‐driven pathology is discussed, introducing current concepts and the underlying experimental evidence. Different aspects of pathological tau phosphorylation, the protein's genomic and domain organization as well as its spreading in disease, together with *MAPT*‐associated mutations and their respective models are presented. Dysfunction related to other post‐transcriptional modifications and their effect on normal neuronal functions such as cell cycle, epigenetics and synapse dynamics are also discussed, providing a mechanistic explanation for the observations made in FTLD‐tau cases, with the possibility for therapeutic intervention.

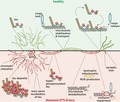

In this review, we cover aspects of tau function, both in the healthy and diseased brain, referring to different *in vitro* and *in vivo* models. In healthy neurons, tau is compartmentalized, with higher concentrations found in the distal part of the axon. Cargo molecules are sensitive to this gradient. A disturbed tau distribution, as found in frontotemporal lobar degeneration (FTLD‐tau), has severe consequences for cellular physiology: tau accumulates in the neuronal soma and dendrites, leading among others to microtubule depolymerization and impaired axonal transport. Tau forms insoluble aggregates that sequester additional molecules stalling cellular physiology. Neuronal communication is gradually lost as toxic tau accumulates in dendritic spines with subsequent degeneration of synapses and synaptic loss. Thus, by providing a mechanistic explanation for the observations made in FTLD‐tau cases, arises a possibility for therapeutic interventions.

**This article is part of the** Frontotemporal Dementia special issue.

**Abbreviations used**
0N or 1N or 2N: no or 1 or 2 N‐terminal repeats domain
2R or 3R: 2 or 3 C‐terminal repeats domain
AAV: adeno‐associated virus
AD: Alzheimer's disease
aFTLD‐U: atypical FTLD with ubiquitinated inclusion
AGD: argyrophilic grain disease
AGE products: advanced glycation end products
ALS: amyotropic lateral sclerosis
AMPA: α‐amino‐3‐hydroxy‐5‐methyl‐4‐isoxazolepropionic acid
APP: amyloid precursor protein
Aβ: amyloid‐β
BIBD: basophilic inclusions body disease
CamKII‐tTA promoter: CaMKll promoter regulating the tetracycline transactivator
CBD: cortical basal degeneration
CDK5: cyclin‐dependent kinase 5
C‐terminal: carboxy‐terminal
Cx3cr1: CX3C chemokine receptor 1
Drp1: dynamin‐related 1
dTau: *Drosphila* tau
ERK: extracellular‐regulated kinase
ESCs: embryonic stem cells
FLIM/FRET: Fluorescence Lifetime Imaging Microscopy/Fluorescence Resonance Energy Transfer
FTD‐3: FTLD linked to chromosome 3
FTDP‐17: FTLD with parkinsonism linked to chromosome 17
FTDP‐17T: FTLD with parkinsonism linked to chromosome 17 with *MAPT* mutations
FTLD: Frontotemporal lobar degeneration
FUS: fused in sarcoma
GFP: green fluorescent protein
*GRN*: progranulin gene
GSK3: glycogen synthase‐3
iPSCs: induced pluripotent stem cells
JNK: c‐Jun N‐terminal kinase
ko: knock‐out
LTD: long‐term depression
LTP: long‐term potentiation
MAP2: microtubule‐associated protein 2
MAP4: microtubule‐associated protein 4
MAPK: mitogen‐activated protein kinase
MAP: microtubule‐associated protein
*MAPT*: microtubule‐associated protein tau gene
MARK: microtubule‐affinity regulating kinase
mRNA: messenger RNA
MSTD: multiple system tauopathy with dementia
mThy1.2 or hThy1.2 promoter: mouse or human Thy1.2 promoter
NDT‐dementia: neurofibrillary tangle predominant dementia
NFT: neurofibrillary tangle
NIFID: neuronal intermediate filament inclusion disease
NREM: non‐rapid eye movement
N‐terminal: amino‐terminal
O‐GlcNAc: O‐linked N‐acetylglucosamine
PAC: P1‐derived artificial chromosome
Par1: protease‐activated receptor 1
PDGFβ: platelet‐derived growth factor β‐chain
PD: Parkinson's disease
PHF: paired helical filaments
PiD: Pick's disease
PolyI:C: polyriboinosinic‐polyribocytidilic acid
PP1A or PP2A: protein phosphatase 2A or 2B
PrP: prion protein promotor
PSD‐95: post‐synaptic density protein 95
PSEN1: presenilin 1 gene
PSP: progressive supranuclear palsy
Ptl‐1: protein with tau‐like repeats‐1
PXXP: proline‐rich motif
RNAi: RNA interference
ROS: reactive oxygen species
SAGE: serial analysis of gene expression
SFPQ: splicing factor proline/glutamine‐rich
Sgg: protein kinase shaggy
SH1/2/3: Src homology domain 1/2/3
SLM: stratum lacunosum moleculare
SPR: surface plasmon resonance
STEP: striatal‐enriched protein tyrosine phosphatase
SUMO: small ubiquitin‐related modifiers
*TARDP*: TAR DNA‐binding protein 43 gene
UPS: ubiquitin proteasome system
*VCP*: valosin containing protein gene
WMT‐GGI: white matter tauopathy with globular glial inclusions

Frontotemporal dementia or frontotemporal lobar degeneration (FTLD) is a debilitating disease that results from progressive neurodegeneration and eventually, atrophy of the frontal and temporal cortex as well as subcortical regions. The onset of FTLD is insidious, with heterogeneous progressive clinical features that exhibit at least two of the following three sets of symptoms: behavioural and personality changes, cognitive deficits and Parkinson‐like motor dysfunctions (Ghetti *et al*. 2015).

The first paper describing temporal lobar cortical atrophy is attributed to Arnold Pick (Pick 1892). Subsequently, other neuropathologists made similar observations on frontotemporal atrophy, which differs from the widespread atrophy that characterizes senile dementia. At the turn of the 19^th^ century, an important contribution was made by Alois Alzheimer who described cases of lobar cortical atrophy with intracytoplasmic argyrophilic inclusions and ballooned neurons, without the plaques characteristic of the disease that now carries his name (Alzheimer 1907, 1911). In the 20th century, progress was made to better identify and characterize what would later be named FTLD. The 1950s saw the first comprehensive clinico‐pathological studies of FTLD (Delay *et al*. 1957), and further characterization of FTLD led to a set of criteria to allow a more accurate neuropathologic diagnosis and classification (Cairns *et al*. 2007). A current alternative classification of FTLD is based on the observed changes in behaviour, language, movement and/or cognition caused by atrophy of the frontal and temporal cortex. In this system of cognitive syndromes, FTLD cases with mainly behavioural changes are grouped under the umbrella term of ‘behavioural‐variant FTD’ (Rascovsky *et al*. 2011), whereas those with language deficits are classified as primary progressive aphasias that are further subdivided into semantic dementia and progressive non‐fluent aphasia (Gorno‐Tempini *et al*. 2011). A genetic component of FTLD has also been identified, with approximately 40% of affected patients being reported to have some form of family history of FTLD or dementia, but only 10% of cases being dominantly inherited (Goedert *et al*. 2012). Recently, Mackenzie and colleagues up‐dated the previously established classification of FTLD based on the specific pattern of pathology (Mackenzie *et al*. 2010). Accordingly, a first molecular group was defined as FTLD‐tau, characterized by tau deposits and encompassing Pick's disease (PiD), cortical basal degeneration (CBD), progressive supranuclear palsy (PSP), argyrophilic grain disease (AGD), multiple system tauopathy with dementia, neurofibrillary tangle predominant dementia (NFT‐dementia) and white matter tauopathy with globular glial inclusions. Further categories are FTLD‐TDP (associated with deposits of TDP‐43), FTLD‐UPS (linked to chromosome 3), FTLD‐FUS and FTLD without overt inclusions. However, this classification is not absolute and has its limitations. For example frontotemporal dementia with parkinsonism linked to chromosome 17 (FTDP‐17) is not included since it is not characterized by a consistent pattern of pathology (Mackenzie *et al*. 2010).

## Tau and FTLD‐tau

Tau protein inclusions are the hallmark of neurodegenerative diseases collectively referred to as tauopathies (Lee *et al*. 2001). Tau belongs to the family of microtubule‐associated proteins (MAPs), which are found throughout the entire animal kingdom, from *Caenorhabditis elegans* and *Drosophila melanogaster* to rodents, non‐human primates and humans (Buée *et al*. 2000; Dehmelt and Halpain 2005). The MAPs have evolved to fulfil various cellular functions: as microtubule motors, centrosome‐associated proteins, enzymatically active MAPs and structural MAPs with scaffolding function. In mammals, the latter category is represented by the neuronal proteins microtubule‐associated protein 2 (MAP2), tau and the non‐neuronal protein microtubule‐associated protein 4 (Dehmelt and Halpain 2005).

The study of tau began in the 1970s, when Weingarten and colleagues described a protein that is essential for the assembly of microtubules, a cytoskeletal element composed of polymers of tubulin, and named it tau (tubulin‐associated unit) or τ (Weingarten *et al*. 1975). The following decade saw the initial identification of the tau‐encoding microtubule‐associated protein tau gene (*MAPT*) gene (Drubin *et al*. 1984; Goedert *et al*. 1988). The protein tau was found to bind to the carboxy‐terminal region of tubulin through a region known as the microtubule‐binding domain (Goedert *et al*. 1989), and it was reported to localize to axonal microtubules in the healthy brain, whereas under pathological conditions it is abnormally concentrated in a hyperphosphorylated form in the somato‐dendritic compartment of neurons (Kosik *et al*. 1986; Wood *et al*. 1986). Hyperphosphorylated forms of tau were also found to constitute the major proteinaceous component of the neurofibrillary tangles (NFTs) found in Alzheimer's disease (AD) brains, and of the Pick bodies found in PiD (Grundke‐Iqbal *et al*. 1986). In the 1990s, familial forms of FTLD were linked to chromosome 17q21–22 (Wilhelmsen *et al*. 1994; Murrell *et al*. 1997) and named FTDP‐17, culminating in the identification of pathogenic mutations in *MAPT* (Hutton *et al*. 1998; Poorkaj *et al*. 1998; Spillantini *et al*. 1998b). Today, it is accepted that FTLD associated with *MAPT* mutations is a highly heterogeneous disorder that affects behaviour, language, memory and motor functions. FTLD‐tau often begins with psychiatric symptoms and has similarities to other neurodegenerative diseases, such as AD (Spillantini and Goedert 2013). The diagnosis of FTLD‐tau can be challenging when based solely on a clinical or histopathological assessment, as tau inclusions are present in all aforementioned neurodegenerative diseases (Cairns *et al*. 2007; Goedert 2015).

## Tau function in the healthy human brain

**Figure 1:**
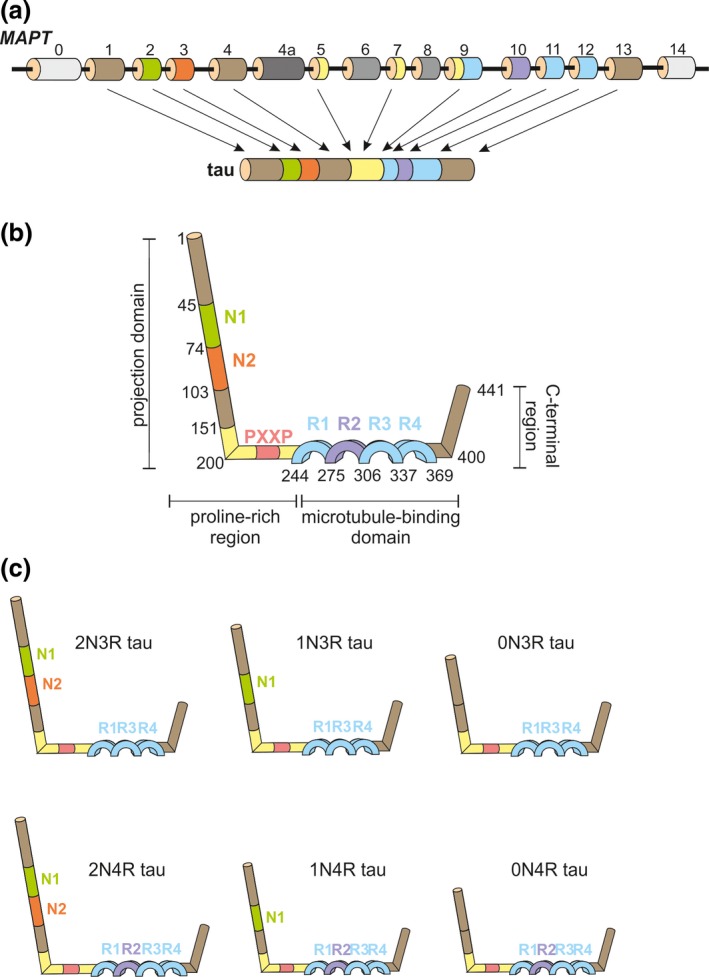
Structure of human microtubule‐associated protein tau gene (*MAPT*) and protein. (a) *MAPT* contains 16 exons. In the brain, 11 of these exons are alternatively spliced, leading to the expression of different tau isoforms. Exons 4a, 6 and 8 are insufficiently studied. The exons are colour‐coded matching the corresponding tau protein domains; (b) The general structure of the longest human tau protein encompasses an N‐terminal projection domain, a proline‐rich region, a microtubule‐binding domain and a C‐terminal region. The N‐terminal region can contain acidic regions (N1, N2), followed by a proline‐rich region bearing up to seven PXXP motifs that can interact with other proteins via Src‐homology 3 (SH3) domains. Preceding the C‐terminal region, tau possess a microtubule‐binding domain composed of tubulin‐binding repetitions (R1, R2, R3, R4); (c) The principal 6 isoforms of human tau that are expressed in brain contain a common backbone represented by three tubulin‐binding repeats (R1, R3 and R4), to which either N1, N1 together with N2 or none of these acidic domains are added, resulting in the 2N3R, 1N3R or 0N3R isoforms. Inclusion of exon 10 of *MAPT* leads to the presence of the R2 tubulin‐binding domain, resulting in the 2N4R, 1N4R or 0N4R tau isoforms.

Tau is an intrinsically disordered protein, with a structure that can be subdivided into four domains: N‐terminal, proline‐rich, microtubule‐binding and C‐terminal (Fig. 1). Tau is rich in polar charged amino acids, displaying a generally basic character, except for the first 120 amino acids that are acidic (Mandelkow and Mandelkow 2012). Interestingly, about 50% of the sequence comprises only five amino acids: glycine, lysine, proline, serine and threonine. The single gene encoding tau, *MAPT*, is found on chromosome 17q21–22 and consists of 16 exons (Fig. 1a). In Caucasians, there are two haplotypes of *MAPT* resulting from a 900 kb inversion (H1) or non‐inversion (H2) polymorphism (Baker *et al*. 1999; Stefansson *et al*. 2005), with the former increasing the risk of neurodegenerative diseases such as PSP (Baker *et al*. 1999; Höglinger *et al*. 2011), CBD (Cruchaga *et al*. 2009), amyotrophic lateral sclerosis or Parkinson's disease (Sundar *et al*. 2006).

The constitutively expressed exons of *MAPT* are 1, 4, 5, 7, 9, 11 and 12, whereas exons 2, 3 and 10 are alternatively spliced, giving rise to six major adult brain‐specific isoforms, ranging from 352 to 441 amino acids (Fig. 1) (Goedert *et al*. 1992). Exons 4A, 6 and 8 have not been identified in any human brain mRNA at any developmental stage, even though exon 4A is expressed in peripheral nerves (Georgieff *et al*. 1993). Exon 6‐containing transcripts have been insufficiently studied to date (Wei and Andreadis 1998). The N‐terminus or so‐called projection domain is not instrumental in binding to microtubules (Fig. 1b). It consists of highly acidic inserts represented by expression of either exon 2 alone (1N, 29 amino acids), exons 2 and 3 (2N, 58 amino acids in total) or neither of them (0N; Fig. 1c). These inserts are followed by a basic, proline‐rich region. The C‐terminal half of tau is composed of tubulin‐binding motifs, represented by either three or four repeat‐domains (3R or 4R respectively; Fig. 1c), and a C‐terminal tail. The presence of the repeat motifs correlates with the capacity of tau to bind, and thus stabilize, microtubules, with 4R increasing the interaction with microtubules (Goedert *et al*. 1989; Goedert and Jakes 1990).

During human brain development, 3R tau predominates, whereas in the adult brain, 3R and 4R are found in an approximately equimolar ratio, both at the mRNA and protein level (Avila 2009). Changes to this ratio can lead to neurodegeneration and dementia: in the case of PSP and CBD, the 4R isoform predominates, whereas in PiD, the 3R tau is majoritarly expressed (Buée *et al*. 2000). Interestingly, neuronal subpopulations differ in their isoform expression, e.g. granular cells of the hippocampal dentate gyrus lack mRNAs containing exon 10, leading to the absence of 4R tau (Goedert *et al*. 1989). Moreover, in the retina and peripheral neurons with long axons, an additional high molecular weight tau isoform, called ‘big tau’ has been identified, characterized by the expression of exon 4A (Georgieff *et al*. 1993). Together, these findings suggest a role for cell‐specific tau isoforms in assuming distinct physiological roles.

**Figure 2:**
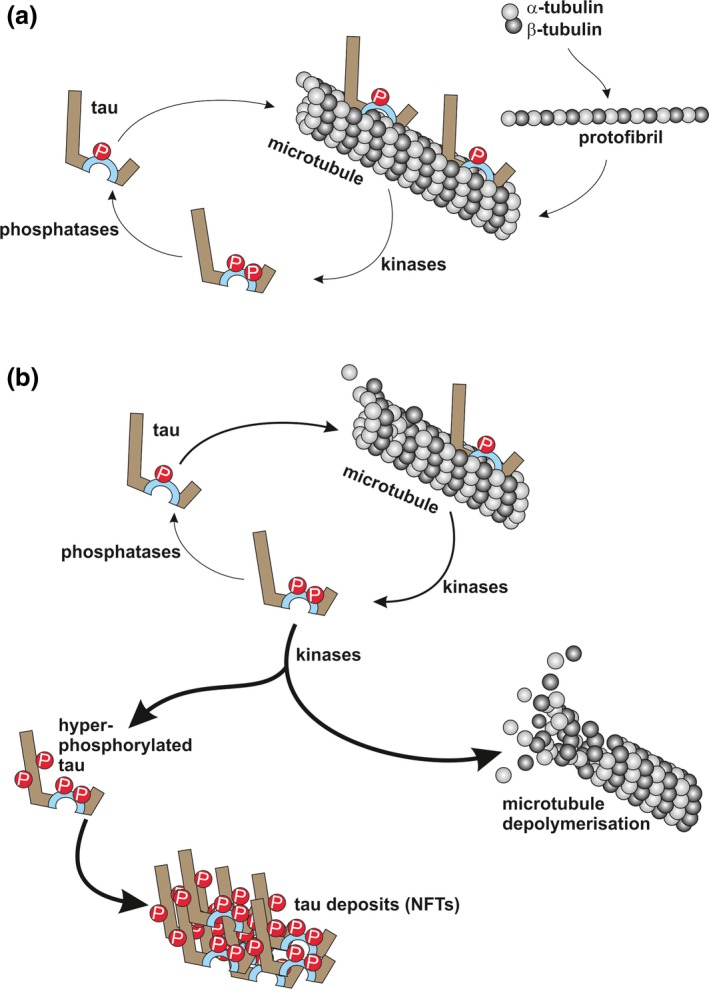
Tau‐microtubule dynamics under healthy and pathological conditions; (a) Heterodimers of α‐ and β‐tubulin assemble into protofibrils, which then form microtubules. Tau can interact with microtubules in a physiologically phosphorylated form. Increased phosphorylation of tau (e.g. as a consequence of glycogen synthase‐3, GSK3, activity) results in detachment from microtubules. Tau is reintroduced in the cycle by the action of protein phosphatases (e.g. PP2A); (b) Hyperphosphorylation of tau sequesters it from its physiological cycle (thicker arrow), resulting in the formation of intracellular tau deposits and eventually neurofibrillary tangles (NFTs), and an increasing microtubule break‐down (thickest arrows).

By binding to microtubules, which are formed by protofibrils of α‐ and β‐tubulin heterodimers, it has been postulated that tau confers structural stability to the microtubules (Weingarten *et al*. 1975). In this model (Fig. 2a), the tubulin‐binding repeats recognize specific sites on the protofilaments in either a cis‐ or trans‐configuration, thereby contributing to microtubule assembly, whereas the positively charged proline‐rich regions are tightly bound to the negatively charged surface of the protofibrils and the negatively charged N‐terminal domain projects further away from the microtubule surface, possibly because of electrostatic repulsion (Kar *et al*. 2003; Santarella *et al*. 2004). Because tau's projection domain determines the distance between axonal microtubules as observed in an insect cell model (Chen *et al*. 1992), it seems likely that the N‐terminal region of tau is crucial for the organization of axons (Buée *et al*. 2000).

Notably, there is a different organization between axons and dendrites in regards to microtubules and the presence of MAP2/tau. Axonal microtubules have a uniform orientation, with the active polymerizing end positioned towards the distal extremity, contributing to axonal specification (Tanaka and Kirschner 1991). The axon is also presenting the highest concentration of tau, with a gradient towards its distal part (Black *et al*. 1996). In contrast, dendritic microtubules have a heterogeneous orientation and although tau is also found in dendrites, albeit at lower levels, MAP2 is fully confined to this compartment (Xia *et al*. 2015). A biochemical study revealed the possibility of microtubule stabilization by an electrostatic zipper‐like dimerization of tau, with the highly negatively charged N‐terminal domain of one tau molecule interacting with the highly positively charged proline‐rich region of an adjacent tau molecule (Rosenberg *et al*. 2008). This model may explain the tight arrangement of microtubules in axons.

A recent study in neuronal cells was able to visualize and characterize the dynamics of tau molecules, proposing a new fast‐paced ‘kiss‐and‐hop’ mechanism (Janning *et al*. 2014). In this report, the interaction of tau with microtubules was shown to last for only ≈40 ms rather than ≈4s, as was previously thought (Konzack *et al*. 2007). Furthermore, the seemingly undirected trajectory of tau movements did not interfere with the normal physiological polymerization of microtubules (Janning *et al*. 2014). These findings seem to be in concordance with the *in vitro* and *in vivo* findings of another group, that observed that the population of tau‐bound microtubules has the highest turnover rate when compared with any other MAPs (Fanara *et al*. 2010). Based on this, the initial postulate that tau confers stability to microtubules seems questionable or at least incomplete (Morris *et al*. 2011). Besides microtubules, tau can also bind other molecules (for review see Morris *et al*. 2011; Mandelkow and Mandelkow 2012). Interestingly, tau interacts with actin, as demonstrated both *in vitro* (Zmuda and Rivas 2000; Sharma *et al*. 2007) and *in vivo* (Fulga *et al*. 2007). This interaction is also mediated *in vivo* by the microtubule‐binding domain, possibly contributing to a cytoskeleton‐associated function (He *et al*. 2009).

## Tau in model organisms

Historically, tauopathy‐related mechanisms were derived from *in vitro* biochemical studies or observations on animal models. For example whereas the initial studies observed the role of tau in microtubule assembly *in vitro*, on samples obtained from porcine or chicken brains (Weingarten *et al*. 1975), the identification of the *Mapt* gene was initially achieved in mice (Lee *et al*. 1988) and 1 year later in humans (Goedert *et al*. 1989). This bottom‐up approach has the obvious advantage of achieving a step‐wise mechanistic understanding, which is unattainable from studying the end‐stage of human disease in *post‐mortem* brain tissue. However, the knowledge obtained from non‐human samples or models provides the foundation for understanding the complexity of the interactions and functions of tau in both the healthy and diseased brain.

The first unambiguously characterized functional orthologue of the MAP2/tau‐family in the evolutionary chain was identified in the *Caenorhabditis elegans* worm model and termed Protein with tau‐like repeats‐1, Ptl‐1 (Goedert *et al*. 1996; McDermott *et al*. 1996). *C. elegans* has a nervous system comprising 302 neurons and as such has been used extensively to model human neurodegenerative diseases (Calahorro and Ruiz‐Rubio 2011). Ptl‐1 presents with a 50% similarity to its mammalian homologue and has two alternatively spliced forms, Ptl‐1A and Ptl‐1B, with four or five microtubule‐repeat domains respectively. Ptl‐1 is neuronally expressed and associates with homologues of the motor proteins kinesin and dynein (Tien *et al*. 2011). Interestingly, a knock‐out (ko) of Ptl‐1 does not affect tubulin organization (Gordon *et al*. 2008), but induces neuronal ageing and a shorter lifespan because of oxidative stress‐related mechanisms (Chew *et al*. 2013, 2015) or altered O‐linked N‐acetylglucosamine (O‐GlcNAc) cycling (Hanover and Wang 2013). Pan‐neuronal *unc‐119* promoter‐driven expression of different tau species, such as human 0N3R or 0N4R isoforms, or only the 0N, 3R or 4R domains of tau, as well as MAP2, induced neurotoxicity in *C. elegans* (Xie *et al*. 2014). The same study reported a higher toxicity of 4R compared to 3R tau in the absence of aggregate formation (Xie *et al*. 2014).

The initial paper describing tau in the fly *Drosophila melanogaster* (dTau) reported an endogenous protein with five microtubule‐binding domains resembling those found in mammalian MAP2/tau, and a 66% similarity to the corresponding human tau homologue (Heidary and Fortini 2001). However, this is only the longest, most frequent and most studied of the dTau isoforms. Two other isoforms are characterized by alterations in the C‐terminal region, whereas yet another isoform was found to be entirely absent from adult females (Bouleau and Tricoire 2015). Initially, no obvious morphological or behavioural defects were described in dTau null flies (Doerflinger *et al*. 2003), however, a more recent study re‐examined these flies and observed the presence of isoforms generated from additional internal transcriptional start sites that were not excised in the previous ko approach, thereby explaining the described lack of phenotype (Bolkan and Kretzschmar 2014). Complete elimination of all dTau isoforms resulted in developmental lethality, whereas a knock‐down approach led to progressive eye and central nervous system degeneration (Bolkan and Kretzschmar 2014). Moreover, a partial compensatory effect of Map1b was demonstrated in the dTau knock‐down flies, which was rescued when human tau was expressed (Bolkan and Kretzschmar 2014). A shorter lifespan and neurodegenerative vacuolization in the absence of neuronal tau aggregates were reported in flies expressing human tau (Wittmann *et al*. 2001). Over‐expression of a single copy of dTau or human tau in *D. melanogaster* led to a rough eye phenotype (Chen *et al*. 2007), indicative of impaired cell proliferation, growth, cell determination or differentiation during eye development (Gonzalez 2013). Moreover, introduction of additional tau copies resulted in a gradually increased neurotoxicity in the same model (Chen *et al*. 2007). This could indicate that tau levels need to be balanced to prevent neurodegeneration. On the other hand, in the absence of time‐dependent vacuolization of the optic lobes and loss of photoreceptors, these findings could simply reflect a tau‐related developmental deficit (Sun and Chen 2015). To add to this complexity, Kosmidis and colleagues described ablation of the mushroom bodies after pan‐neuronal accumulation of either 0N4R or 2N4R human Tau, but no effect on the mushroom bodies when dTau, bovine tau or 0N3R human tau were expressed, even though deficits of learning and memory were present (Kosmidis *et al*. 2010). Together, these data support both a structural and a functional similarity between human tau and dTau; they also suggest the presence of species‐specific features that cannot be compensated by homologous molecules.

Zebrafish (*Danio rerio*) has not been widely used to investigate tau‐related functions or tauopathies. Only recently, tau homologues were analysed in zebrafish, revealing the presence of two *MAPT* paralogues, *mapta* and *maptb*, resulting from the duplication of an ancestral teleost *MAPT* orthologue (Chen *et al*. 2009). Interestingly, *mapta* leads to splice variants that have either four or six microtubule‐binding repeats, whereas *maptb* is mainly spliced into a 3R isoform (Chen *et al*. 2009). The first tau zebrafish model expressed human 4R tau fused to green fluorescent protein under a neuron‐specific variant of the *gata2* promoter (Tomasiewicz *et al*. 2002). The model was characterized by cytoskeletal disruptions and alterations in tau trafficking, with tau accumulating in the cell body (Tomasiewicz *et al*. 2002). A subsequent study expressed human 4R tau in zebrafish under the transcriptional control of the *eno2* promoter, reporting a widespread expression of tau throughout the brain, although it was confined to axons and cell bodies (Bai *et al*. 2007).

With no doubt, rodents (mice, *Mus musculus*, or rats, *Rattus norvegicus*) represent the most widely used *in vivo* models of neurodegeneration. The murine tau sequence is 89% identical and 92% similar to that of humans (Morris *et al*. 2015). Seminal studies have shown that, as in humans, foetal brains of both mice and rats express the shortest tau isoform, 0N3R, but, unlike humans, the adult rodent brain predominantly expresses 4R tau (Kosik *et al*. 1989; Kampers *et al*. 1999; Liu and Götz 2013). Mouse studies have demonstrated the presence of tau compartmentalization in neurons, with a high concentration of tau in axons (Ittner *et al*. 2010; Zempel and Mandelkow 2014; Xia *et al*. 2015). Moreover, as revealed in murine neurons, there is an additional intraneuronal compartmentalization: 0N isoforms are mainly found in cell bodies and axons and at lower levels in nuclei and dendrites; 1N isoforms are enriched in the soluble nuclear fraction, although they are also found in cell bodies and dendrites, but not axons, and 2N isoforms are highly expressed in axons and in cell bodies, at a lower level in dendrites and with only a very slight expression in nuclei (Liu and Götz 2013). Furthermore, murine tau shows a higher degree of polymorphism in the N‐terminal domain in comparison with human tau, whereas the C‐terminal domain and its binding regions are almost identical, except for three amino acid residues (Kampers *et al*. 1999). As in humans, ‘big tau’ isoforms have been found in the peripheral nerves of both mice (Couchie *et al*. 1992) and rats (Boyne *et al*. 1995).

In primary neuronal cultures from rat, blocking tau expression with antisense oligonucleotides suppresses axonal elongation (Caceres and Kosik 1990). In 1994, the first tau ko mouse was generated by replacing the first coding exon of tau with a neomycin selection cassette (Harada *et al*. 1994). In these mice, no overt phenotype was encountered, neither *in vivo* nor *in vitro*, possibly because of a compensatory increase in *Map1a* expression (Harada *et al*. 1994). However, when both tau and Map1b were knocked out, this resulted in defects in axonal elongation and neuronal migration (Takei *et al*. 2000). In the following decade, additional tau ko strains were generated by conventional techniques, all of which were phenotypically normal up to 7–8 months of age, generally with a compensatory increase in Map1a around birth that decreased with age (Dawson *et al*. 2001; Tucker *et al*. 2001). Studies of some of these ko strains revealed both behavioural changes and motor deficits with advanced age (Ikegami *et al*. 2000; Lei *et al*. 2012; Ma *et al*. 2014), proving that even when compensatory mechanism were able to maintain normal brain development, they could not substitute for all tau‐specific functions throughout the entire lifespan. Interestingly, the tau ko mice generated by Dawson and colleagues, kept on a C57BL/6/SV129 mixed genetic background, showed reductions in dopaminergic neurons (Lei *et al*. 2012), suggesting that the absence of tau or its dysfunction can act as a neurodegenerative trigger for this particularly sensitive neuronal population (Lannuzel *et al*. 2003; Bodea *et al*. 2014). However, a subsequent study of the same mouse line kept on the original background found normal age‐related cognition and only subtle dopamine‐independent motor deficits (Morris *et al*. 2013). Another study found increased levels of Map1a, Map1b and Map2 in 8–9 month‐old tau ko animals, followed by a loss of Map1b and Map2 in 19–20‐month‐old mice (Ma *et al*. 2014). Moreover, at 19–20 months, but not at a young age, these mice presented memory deficits and loss of acetylated α‐tubulin in the hippocampus, together with the loss of excitatory synaptic proteins (Ma *et al*. 2014). Behavioural studies reported increased wakefulness and decreased non‐rapid eye movement sleep time, a higher number of state transitions between non‐rapid eye movement and wakefulness, and shortened sleep bouts, linking tau to the regulation of the sleep‐wake cycle (Cantero *et al*. 2010a). Moreover, electrophysiology revealed anomalies characteristic of dysfunctional neuronal networks in the Dawson tau ko strain, suggesting a role for tau in forming collective neural responses during the generation of the hippocampal theta rhythm, as well as in the formation of functional circuits between the frontal cortex and other brain regions through gamma oscillations (Cantero *et al*. 2011).

Another study using murine tau ko cultures showed impaired neuronal maturation, confirming previous reports (Caceres and Kosik 1990), and observed that the phenotype was rescued by expressing human tau (Dawson *et al*. 2001). Using a knock‐in approach, Sennvik and colleagues were able to introduce human 2N4R tau into the endogenous tau locus in mice (Sennvik *et al*. 2007). These mice presented enlarged hippocampi because of increased neurogenesis, mirrored by an improved performance in the novel object recognition task. Primary neuronal cultures from these animals also displayed a delayed maturation, suggesting that human tau is not able to substitute for endogenous tau at early stages of brain development.

To study how tau regulates the attachment or detachment of motors involved in the microtubule‐dependent transport of single molecules, cells were stably transfected with tau constructs containing the microtubule‐binding domain, with or without the projection domain (Trinczek *et al*. 1999). It was found that tau perturbed the balance of the bidirectional motion of vesicles by decreasing the reversal frequency from minus‐end‐ to plus‐end‐directed transport. These observations suggested that tau does not affect motor activity itself, but rather acts as a ‘speed bump’, that halts or diverts transport, and as an inhibitor of motor attachment which preferentially affects kinesin (Trinczek *et al*. 1999). In subsequent studies, even though tau was found to be involved in microtubule assembly, the velocity of axonal transport seemed not to be altered in the tau ko mice generated by Tucker and colleagues compared to wild‐type controls (Yuan *et al*. 2008). However, in an elegant *in vitro* study it was revealed that motor proteins involved in cargo transport are dependent on the presence of a tau concentration gradient (Dixit *et al*. 2008). Thus, at the lower levels of tau, as found in the neuronal cell body and proximal parts of the axon, kinesin efficiently binds to microtubules and initiates anterograde transport of cargos, whereas the release of cargos is facilitated at the distal synapse, where a higher tau concentration is present (Dixit *et al*. 2008). In contrast, dynein‐driven retrograde transport away from the distal axon would not be impeded because of dynein's lower sensitivity to tau (Dixit *et al*. 2008).

Taken together, studies in animal models have led to a better understanding of the structure and function of tau, providing insight into tau‐dependent pathogenesis.

## Tau‐driven pathological mechanisms

Tau phosphorylation has been implicated in the pathogenesis of FTLD and other tauopathies during the 1980s (Grundke‐Iqbal *et al*. 1986). In the following decade, pathogenic mutations in the *MAPT* gene were discovered in familial cases of FTLD (Hutton *et al*. 1998; Poorkaj *et al*. 1998; Spillantini *et al*. 1998b). These mutations either alter the physical properties of tau or its 3R/4R isoform ratio. Moreover, extracellular factors impact on tau pathology, as is evident in AD, where extracellular amyloid beta (Aβ) deposits trigger intraneuronal tau‐dependent pathological processes (Ittner *et al*. 2010). In fact, a significant fraction of the knowledge on tau pathomechanisms is derived from studies in AD models, and it is becoming increasingly clear that tau pathology is highly complex and dynamic. Moreover, even under physiological conditions and in addition to phosphorylation, tau can undergo post‐translational modifications that include ubiquitination, nitration, acetylation, oxidation and others (Morris *et al*. 2015). How these post‐translational modifications affect tau function is still incompletely understood.

## Tau phosphorylation and neurofibrillary tangle formation

Tau phosphorylation is pronounced in the developing brain and reduced in the healthy adult brain (Morishima‐Kawashima *et al*. 1995). However, even though many of its phosphorylation sites that are affected in both tauopathies and relevant animal models are known today (for an exhaustive list, see the online resources of Dr Diane Hanger's laboratory at http://cnr.iop.kcl.ac.uk/hangerlab/tautable/), little is known about the actual role of distinct phosphorylation events under physiological conditions. Initially, it was observed in cell lines that tau phosphorylation leads to its detachment from microtubules (Trinczek *et al*. 1999), which was explained as either leading to the neutralization of the positive charge of the microtubule‐binding domain as shown *in silico* (Jho *et al*. 2010), conformational changes in the same domain following phosphorylation as shown biochemically (Fischer *et al*. 2009), or a combination of both. Besides its obvious role in microtubule dynamics, phosphorylation of tau seems to have additional roles, as revealed in a study in rats and mice, in which tau phosphorylation enabled neurons to escape from acute apoptotic death through the stabilization of β‐catenin (Li *et al*., 2007). It is also worth mentioning that, besides neuronal pathology, in both familial and sporadic cases of FTLD‐tau, tau inclusions have also been reported in glia (LoPresti *et al*. 1995; Klein *et al*. 2002; Kovacs *et al*. 2008). Glial tau pathology is prominent and it can be more pronounced than neuronal pathology, a fact that is often overlooked.

It has been hypothesized that tau in its normal phosphorylated state contributes to microtubule recycling or other cellular functions, whereas hyperphosphorylation sequesters tau, leading to conformational changes and ultimately, aggregation (Fig. 2). Even though it is acknowledged that hyperphosphorylated tau is found accumulated in deposits (Grundke‐Iqbal *et al*. 1986; Bancher *et al*. 1989), the mechanisms responsible for this tau aggregation are not fully understood. The formation of NFTs is thought to be a gradual process, starting with the oligomerization of non‐bound hyperphosphorylated tau into pretangles, with a change in β‐sheet conformation, building up filaments such as the paired helical filaments (PHFs) and, finally, leading to assembly into NFTs (Ballatore *et al*. 2007). The role that different forms of tau aggregates have in disease has been reviewed recently, indicating that hyperphosphorylated oligomeric tau is involved in synaptic loss, whereas granular tau oligomers seem to be responsible for neuronal loss (Takashima 2013). To address this, the development of antibodies that can discriminate these different assemblies is currently in high demand. In addition to PHFs, straight and narrow‐twisted filaments have also been described in tauopathies (Goedert *et al*. 1988; Wischik *et al*. 1988).

Tau has 45 serine, 35 threonine and 5 tyrosine residues in the longest 2N4R isoform that can be potentially phosphorylated (Goedert *et al*. 1989). It is thought that one reason for the abnormal increase in tau phosphorylation might be an imbalance in the activity or regulation of kinases and phosphatases. Among the kinases, glycogen synthase‐3 (GSK3), cyclin‐dependent kinase 5, the MAPK family composed of p38, extracellular‐regulated kinase and c‐Jun N‐terminal kinase, and microtubule‐affinity regulating kinase (MARK) have been shown to have a role and are considered as candidates for therapy (reviewed in Lee *et al*. 2011; Tell and Hilgeroth 2013; Medina and Avila 2015). Interestingly, tau phosphorylation pathways seem to be evolutionarily conserved, as revealed by a study of 2N4R human tau over‐expression in *Drosophila*, that described tau hyperphosphorylation via the involvement of the GSK3 homologue shaggy or Sgg (Jackson *et al*. 2002). Generating tau mutants in flies that are resistant to phosphorylation suggested that no specific residue plays a leading role in controlling a tau‐related pathology; rather serine‐/threonine‐proline sites work together towards neurodegeneration (Steinhilb *et al*. 2007). However, in other *Drosophila* studies, the protease‐activated receptor 1, the homologue of MARK, was shown to play an important role in initiating tau phosphorylation (Nishimura *et al*. 2004). Moreover, Sgg seems to be more potent than protease‐activated receptor 1 in phosphorylating tau (Chatterjee *et al*. 2008), suggesting the presence of an hierarchy of tau kinases.

Phosphorylation of tau can be decreased by phosphatases, in particular PP2A and PP2B (Gong *et al*. 2000). PP2A is the major brain phosphatase of tau (Gong *et al*. 2000), and reduced PP2A activity was found to cause hyperphosphorylation and compartmentalization of tau in mice (Kins *et al*. 2001). However, therapeutic modulation of PP2A is challenging because of its multimeric structure and the different intracellular signalling pathways in which it has a role (Kamat *et al*. 2014; Medina and Avila 2015).

The extent to which NFTs contribute to neuropathology is not fully resolved, and recent findings point towards a neurotoxic role of less studied intermediate forms of hyperphosphorylated oligomeric tau (Iqbal *et al*. 2010; Kumar *et al*. 2014). This is because neuronal loss precedes or is independent of NFT formation (Gómez‐Isla *et al*. 1997), with NFT‐positive neurons being claimed to survive for an extended period of time (Morsch *et al*. 1999). Interestingly, during the hibernation‐wake cycle of the European ground squirrel (*Spermophilus citellus*), the formation of PHF‐like structures, in the absence of NFT formation, is a fully reversible process associated with hippocampal neuronal plasticity (Arendt *et al*. 2003). Hyperphosphorylated tau has been found in several species including the spectacled bear, bison, guanaco, Campbell's guenon, rabbit, reindeer, baboon and rhesus monkey, with the two primate species also displaying NFTs (Härtig *et al*. 2000; Schultz *et al*. 2000). However, the more widely used experimental animal models (such as mice and rats) do not show pathological accumulations of tau, even at old age. Nevertheless, models of endogenous tau hyperphosphorylation have been achieved by various techniques, ranging from the use of specific phosphorylation‐inducing chemicals, to the generation of transgenic mice that are characterized by hyperphosphorylated tau aggregates.

Several studies reduced the activity of PP2A chemically, by okadaic acid application (reviewed by Medina *et al*. 2013). In an *in vitro* study, the usage of okadaic acid revealed that tau phosphorylation can actually regulate sequestration of tau within the axon (Li *et al*. 2011). The study explained this observation by the presence of a retrograde barrier mechanism localized at the boundary between the neuronal soma and the axon, at the so‐called axonal initial segment (Li *et al*. 2011). This semi‐permissive barrier allows tau to enter the axon, but prevents it from flowing back into the somato‐dendritic compartment, unless it is phosphorylated at specific sites (Li *et al*. 2011). Human brain slices incubated in the presence of okadaic acid developed PHFs (Harris *et al*. 1993), and injection into the sheep cortex induced immunoreactive dystrophic neurites (Nelson and Saper 1996). Whereas intracerebral injection in rats induced pathological tau phosphorylation (Gärtner *et al*. 1998), intrahippocampal injection led to spatial cognitive deficits and astrogliosis (Costa *et al*. 2012).

Interestingly, alterations in the normal production and/or function of tau can also drive an imbalance in the ratio of normal and hyperphosphorylated forms of tau. This became also evident when the first tau transgenic mouse was generated (Götz *et al*. 1995). In this model, the expression of the longest human tau isoform (2N4R) driven by the neuron‐specific promoter mThy1 was able to recapitulate key features of tauopathies, such as the presence of hyperphosphorylated tau mislocalized to neuronal cell bodies and dendrites (Götz *et al*. 1995). However, NFTs were not detected. Similar findings were obtained in a more advanced model, ALZ17, where the expression of human 2N4R under the control of the stronger hThy1.2 promoter led to signs of Wallerian degeneration and neurogenic muscle atrophy (Probst *et al*. 2000).

The expression of the shortest form of tau (0N3R), this time under the control of the prion protein promoter (PrP), resulted in an age‐dependent pathology as evident by hyperphosphorylated tau inclusions in the cortex and brain stem, gliosis and a generally more aggressive phenotype in the spinal cord (Ishihara *et al*. 1999, 2001b). This model was used by the same group to investigate a possible contribution of neurofilaments to the formation of NFTs by crossing the mice with lines that lack neurofilaments (Ishihara *et al*. 2001a). The resulting cross revealed an attenuated neurodegenerative phenotype in the presence of tau over‐expression, leading to the conclusion that neurofilaments might act as chaperones in the formation of NFTs (Ishihara *et al*. 2001a).

Endogenous murine tau seems to protect from human tau‐induced pathology, suggesting a dominant function of this form of tau in mice. This hypothesis was confirmed by over‐expressing 3R human tau driven by the tubulin‐α1 promoter in the tau ko mice generated by Dawson and colleagues, shifting the accumulation of abnormal tau aggregates into astrocytes and oligodendrocytes, but not neurons, together with a loss of both glial cells and neurons and progressive motor disturbances such as weakness and dystonia (Higuchi *et al*. 2002). Although, tau‐related glial pathology has been described in tauopathies such as PSP, CBD and PiD (Feany and Dickson 1995; Kovacs *et al*. 2008), what causes cell‐specific accumulation is not known. Generating mice with a P1‐derived artificial chromosome containing the coding sequence, intronic regions and regulatory regions of the human tau gene resulted in the expression of all six human tau isoforms in the mouse brain, in the absence of an overt neuropathology (Duff *et al*. 2000). Interestingly, after breeding these P1‐derived artificial chromosome tau transgenic mice onto a tau ko background, tau hyperphosphorylation and translocation in the somato‐dendritic compartment were achieved as early as 2 months of age (Andorfer *et al*. 2003), resulting in neuronal loss together with ventricle enlargement and reduced cortical thickness (Andorfer 2005), memory deficits and perturbed long‐term potentiation at older age (Polydoro *et al*. 2009).

Intraperitoneal injection of mice with recombinant human tau protein led to NFT formation in both neurons and glia, accompanied by clinical symptoms reminiscent of induced experimental autoimmune encephalomyelitis, i.e. neurologic deficits and mononuclear infiltration (Rosenmann *et al*. 2006). However, this particular study focused on the effects of active tau immunization that used complete Freund's adjuvant together with pertussis toxin in the injection mix. In a more recent study addressing the safety profile of clinical trials, an increased encephalitogenic effect of repeated vaccinations with phosphorylated tau was revealed (Rozenstein‐Tsalkovich *et al*. 2013), establishing a possible correlation with the increased neurodegeneration and tau aggregation. Indeed, a neuroinflammatory influence on the development of tauopathy was revealed by Krstic and colleagues using an entirely new approach, which achieved altered tau phosphorylation and mislocalization after intravenous application of virus‐mimicking polyriboinosinic‐polyribocytidilic acid (PolyI:C) either prenatally or into adult mice (Krstic *et al*. 2012). This would suggest that chronic inflammatory conditions could contribute to the development of tauopathy.

Subcortical stereotaxic injections of full‐length human tau in oligomeric, but not fibrillar or monomer form were also found to be neurotoxic, leading to synaptic and mitochondrial dysfunction, neuronal loss and cognitive decline (Lasagna‐Reeves *et al*. 2011). A study in which human 2N4R tau fibrils were injected into C57BL/6 brain revealed that negatively charged extracellular heparan sulphate proteoglycans might be involved in the uptake of fibrils and the initiation of intracellular tau hyperphosphorylation (Holmes *et al*. 2013).

## MAPT mutations

In addition to the knowledge obtained from studying cases of sporadic FTLD brains and animal models, much about tau physiology and its associated pathology has been derived from observing alterations resulting from *MAPT* gene mutations (for an exhaustive list, see the online resource: http://www.molgen.ua.ac.be/ADMutations/). To date, 53 pathogenic *MAPT* mutations have been reported in approximately 150 families (van der Zee and Van 2014). Exonic mutations of *MAPT* almost exclusively affect exons 9–13, with only two mutations being found in exon 1. The majority of the intronic mutations are clustered in the intron following exon 10, which encodes part of the microtubule‐binding region. Mutations in this hotspot of alternative mRNA splicing have been shown to lead to an increase in 4R tau translation, modifying the physiological 3R/4R ratio and affecting microtubule dynamics and other cellular functions (Hutton *et al*. 1998; Spillantini *et al*. 1998b).

Based on the identification of pathogenic *MAPT* mutations, novel NFT‐forming models were successfully generated, mostly by introducing P301L, P301S, N279K, V337M or R406W mutations (for a more extensive review of the corresponding mouse strains see Götz *et al*. 2011; Dujardin *et al*. 2015; Ballatore *et al*. 2007). Unlike wild‐type tau transgenic animals, most of the models expressing *MAPT* mutations form NFTs within 3–9 months. In 2000, the JNPL3 transgenic strain was generated by expressing the human 0N4R tau isoform bearing the P301L tau mutation under control of the PrP promoter (Lewis *et al*. 2000). The P301L mutation has previously been associated with FTLD‐tau presenting with the classic hallmark of tauopathy – intracytoplasmic hyperphosphorylated tau in multiple brain regions, including the hippocampus, neocortex and substantia nigra (Dumanchin *et al*. 1998; Hutton *et al*. 1998; Spillantini *et al*. 1998a). The JNPL3 strain represented the first mouse model that expressed a mutant form of tau which led to NFT formation at around 4–5 months of age, and progressed to motor dysfunction, neuronal loss and astrogliosis by 10 months (Lewis *et al*. 2000). By expressing the human 2N4R tau isoform bearing the P301L mutation under control of the neuron‐specific promoter mThy1.2, another line, pR5, recapitulated the hallmarks of tauopathy (Götz *et al*. 2001a). Unlike the mouse line generated by the same group that expressed wild‐type human 2N4R (Götz *et al*. 1995), the pR5 mice developed NFTs (Götz *et al*. 2001a). The P301L tau mutation eventually became widely expressed in many tauopathy studies, with similar results recapitulated in other organisms. By expressing this mutant form in *D. rerio*, it was possible to visualize early‐stage tau‐associated pathology, including disease‐specific hyperphosphorylation and conformational changes in tau, as well as neuronal and behavioural abnormalities (Paquet *et al*. 2009). Intriguingly, the larvae developed substantial neurodegeneration within a few days, with a much more rapid progression than observed in the corresponding mouse models (Paquet *et al*. 2009). In *C. elegans*, expressing wild‐type human 1N4R tau together with the P301L or V377M mutation caused progressive uncoordinated locomotion indicative of neuronal dysfunction, as well as hyperphosphorylated tau and neuronal loss enhanced by the presence of mutant tau (Kraemer *et al*. 2003). Interestingly, the uncoordinated locomotion phenotype preceded the peak in tau hyperphosphorylation and neuronal loss, being caused by a pre‐synaptic defect (Kraemer *et al*. 2003). This supports the hypothesis of a more powerful neurotoxic effect of oligomeric tau that could affect axonal transport leading to pre‐synaptic pathological alterations. A more aggressive phenotype was observed in a mouse line expressing P301L mutant human 0N4R tau in the forebrain under the control of a tetracycline‐inducible CamKII‐tTA promoter (Santacruz *et al*. 2005). Analysis of this model revealed NFT formation that was directly dependent on the level of tau expression, with the highest levels found in the Tg4510 mouse line that presented a 13‐fold level of transgenic tau expression compared with endogenous tau (Santacruz *et al*. 2005). The mice displayed mature NFTs starting as early as 2.5 months of age and behavioural alterations were recovered after doxycycline‐induced suppression of transgene expression, even though, surprisingly, NFTs continued to accumulate, suggesting a tau‐associated toxic effect independent of NFT formation (Santacruz *et al*. 2005).

The amino acid residue mutated in tau seems to dictate disease severity, onset and duration. This was observed by comparing human brains with the less severe P301L tau mutation with those carrying the P301S mutation (Sperfeld *et al*. 1999; Allen *et al*. 2002). The mouse models expressing P301S were generated to either express human 1N4R tau in the so‐called PS19 model (Yoshiyama *et al*. 2007) or 0N4R tau (Allen *et al*. 2002), the latter model presenting an enhanced NFT pathology when compared with the P301L‐expressing models generated earlier (Lewis *et al*. 2000; Götz *et al*. 2001a). Although both models are characterized by insoluble tau aggregates, neuronal loss and motor and cognitive deficits, they present with different pathological onsets, with the PS19 line being more severe, most likely because of PrP‐promoter‐mediated higher levels of expression (Allen *et al*. 2002; Yoshiyama *et al*. 2007; Scattoni *et al*. 2010; Takeuchi *et al*. 2011).

Confirming the dominant function of tau, pan‐neuronal expression of wild‐type human tau or tau variants carrying the FTLD‐associated mutation R406W were found to cause retinal degeneration in *Drosophila*, with the mutant forms enhancing the severity of the effects (Wittmann *et al*. 2001). In mice expressing R406W on a human 2N4R tau backbone (RW mouse line), widespread insoluble filamentous tau aggregates were found in neuronal perikarya within the cerebral cortex, hippocampus, cerebellum and spinal cord, correlating with impaired axonal transport (Zhang *et al*. 2004). Interestingly, however, even though the RW mice were expressing mutant tau driven by the mouse PrP promoter and expression levels were 8–10 times higher than endogenous tau, pathology was only initiated at 12 months of age (Zhang *et al*. 2004). An even more delayed pathology was described in another mouse model of R406W expression under the CamKII promoter, in which hyperphosphorylated tau deposits were only observed at 18 months of age (Tatebayashi *et al*. 2002). However, in a mouse model expressing the human R406W mutation, but driven by the Syrian hamster PrP promoter and expressing tau levels equivalent to the endogenous one, the age of hippocampal lesions onset was 6 months (Ikeda *et al*. 2005). In addition, micro‐ and astrogliosis was reported at 10 months, suggesting the possibility of a strain‐related effect resulting in a different age of pathological onset. Intriguingly, an *in vitro* study suggested that the effect of the R406W mutation is not because of the impaired binding of tau to microtubules, but is rather caused by impaired membrane binding, which involves a functional interaction with annexin A2 as a membrane–cytoskeleton linker (Gauthier‐Kemper *et al*. 2011).

Another FTLD‐related mutation, V337M (Hutton *et al*. 1998; Spillantini *et al*. 1998a), was introduced into the human 2N4R tau backbone and expressed in mice under control of the platelet‐derived growth factor β‐chain promoter (Tanemura *et al*. 2001). The resulting Tg214 mouse line expressed the transgene at ten times lower levels than endogenous tau, but NFTs still developed by 11 months of age (Tanemura *et al*. 2001). The FTLD‐related G272V mutant tau (Hutton *et al*. 1998; Spillantini *et al*. 1998a) was also introduced into mice under the PrP‐driven transactivator, leading to accumulation of filamentous tau in oligodendrocytes (Götz *et al*. 2001c).

Tau accumulation in glial cells has been reported in many tauopathies, such as PSP, CBD, PiD or FTDP‐17 with *MAPT* mutations (Kouri *et al*. 2011; Spillantini and Goedert 2013; Yoshida 2014; Irwin *et al*. 2015), or is even as specific marker in the case of globular glial tauopathies (Ahmed *et al*. 2013). However, modelling of these diseases was achieved only in a handful of studies. Specific expression of P301L mutant 1N4R human tau in murine oligodendrocytes was achieved using the 2′,3′‐cyclic nucleotide 3′‐phosphodiesterase promoter, leading to structural disruption of myelin and axons that preceded the emergence of tau inclusions in oligodendrocytes (Higuchi *et al*. 2005). By using the glial fibrillary acidic protein promoter, P301L 1N4R human tau was specifically expressed in mouse astrocytes, resulting in an age‐dependent accumulation of tau inclusions, accompanied by compromised motor functions correlating with an altered expression of the glial glutamate‐aspartate transporter before development of tau pathology (Dabir *et al*. 2006).

Many additional transgenic models carrying FTLD mutations in *MAPT* have been generated (for an extensive review see Götz and Ittner 2008; Dujardin *et al*. 2015; Ballatore *et al*. 2007). By combining mutations, it was possible to obtain an enhanced pathology and/or a decreased age of onset of pathology, as in the case of a P301L/G272V/R406W triple mutation introduced into the 2N4R tau backbone under the Thy1 promoter that led to tau pathology as early as 1.5 months of age together with aberrant lysosomal morphology (Lim *et al*. 2001). In another approach, P301L tau transgenic mice were crossed with a PP2A dominant negative strain, resulting in double‐mutant mice with an accelerated NFT pathology, thereby proving the possibility of genetically modulating tau phosphorylation (Deters *et al*. 2009). Advanced AD models have also provided tools to study the complex interaction or involvement of tau in other pathologies. For example the JNPL3 tau mouse line, when crossed with the Tg2576 mouse line that expresses the human amyloid precursor protein gene together with the so‐called Swedish mutation found in familial cases of AD (APP^sw^ mouse line), developed an enhanced neurofibrillary degeneration, demonstrating a cross‐talk of tau and Aβ in neurodegeneration (Lewis *et al*. 2001). Moreover, by injecting preparations of the Aβ peptide into brains of pR5 mice, the downstream position of tau in a pathological cascade of AD development was revealed (Götz *et al*. 2001b). APP_OSK_‐Tg mice, which carry the E693Δ (Osaka) mutation in *APP* and accumulate Aβ oligomers without forming plaques, exhibited tau hyperphosphorylation at 8 months, but failed to develop NFTs even at 24 months of age (Umeda *et al*. 2014). The same study reported an accelerated NFT formation at 18 months, by cross‐breeding the mice with a second tau mouse line, tau 264, that expressed human tau at low levels and in the absence of any pathology (Umeda *et al*. 2013). Moreover, synapse loss and memory impairment were accelerated in the newly generated line, suggesting that the presence of tau in its human form is critical for NFT formation (Umeda *et al*. 2014)**.** Interestingly, breeding APP mutant APP23 mice with P301L tau mutant mice led to accelerated mortality in offsprings, possibly because of increased excitotoxicity (Ittner *et al*. 2010). The 3xTg‐AD model was generated by combining three mutations, APP^swe^, P301L tau and the M146V mutation in the *PSEN1* gene (that encodes a component of the γ‐secretase complex involved in APP processing), with the resulting mouse model closely recapitulating human AD pathology by forming both Aβ plaques and NFTs (Oddo *et al*. 2003; Rhein *et al*. 2009).

In another line of research, human tau was delivered to mice using viral vectors. When adeno‐associated viral vectors (AAVs) of serotype 1/2 encoding human 2N4R tau were applied diffusely into the hippocampus of wild‐type mice, a dramatic degeneration of pyramidal neurons of the hippocampal CA1/2 region and the cortex was obtained within weeks, in the absence of NFT formation (Jaworski *et al*. 2009). In contrast, injection of serotype 2 AAVs encoding the P301L form of 2N4R tau into Sprague–Dawley rats induced NFT formation 4 months after the injection (Klein *et al*. 2004).

Taken together, these investigations produced different and, at times, even contradictory results, which might be explained by the limitation inherent to each model. However, the transgenic models support the notion that FTLD‐related mutations of *MAPT* enhance tau phosphorylation and cause tau aggregation leading to impaired memory functions and neurodegeneration.

## Other post‐translational modifications of tau

The majority of studies on post‐translational modifications of tau have focused on phosphorylation. However, there are also additional post‐translational modifications of tau that can induce misfolding and subsequent aggregation, thereby affecting normal cellular physiology.

O‐glycosylation has been described as a major form of post‐translational modification that is highly dynamic and responsive to cellular stimuli in a fashion similar to phosphorylation. It is characterized by the addition of an O‐GlcNAc residue on serines or threonines in the proximity of proline residues (Haltiwanger *et al*. 1992; Varki 2007). Given that tau is highly enriched in these amino acid residues, it was expected that it would also be found in an O‐GlcNAc form (Arnold *et al*. 1996), and moreover, that O‐GlcNAcylation might modulate tau function and play a role in its subcellular localization and degradation (Arnold *et al*. 1996). However, more recently, this hypothesis was challenged, as it was found that the number of O‐GlcNAc sites on tau might actually be reduced to just three in the rat brain: T123, S400 and a third which might be either S409, S412 or S413 (Yuzwa *et al*. 2011). Another study of rat tau reduced this number even further, identifying only S400 of tau as being O‐GlcNAc‐modified (Wang *et al*. 2010). This result was subsequently confirmed in mice, although only wild‐type animals were assessed (Morris *et al*. 2015). Increasing the level of O‐GlcNAc tau, by using OGAse inhibitors in JNPL3 hemizygous mice, resulted in a decrease in tau aggregates and neuronal loss, in the absence of alterations in tau phosphorylation, indicating that phosphorylation and O‐GlcNAcylation might not be linked (Yuzwa *et al*. 2012). More recently, interaction of the O‐GlcNAc form of tau with β‐catenin was revealed using a FLIM/FRET‐based imaging approach (Lin *et al*. 2015).

Glycation, also known as ‘non‐enzymatic glycosylation’ or ‘Maillard reaction’, is a process by which sugar molecules are covalently bound to proteins or lipids in a slow process of rearrangements and additions that initially form early glycation adducts, followed by advanced glycation end (AGE) products that are irreversibly modified and tend to aggregate. Tau can suffer glycation reactions in the microtubule‐binding site, which then induces oxidative stress (Ledesma *et al*. 1995). Interestingly, many molecules implicated in AD are associated with or sensitive to binding AGE products (reviewed in Li *et al*. 2012a) and these products were shown to induce tau hyperphosphorylation, resulting in impairments in synapse function and memory in rats (Li *et al*. 2012b).

Ubiquitination, acetylation and methylation are enzyme‐mediated processes. Ubiquitination tags proteins by the addition of ubiquitin, whereas acetylation and methylation are processes by which an acetyl or methyl group, respectively, are transferred from one molecule to another. Thus, the modified proteins become equipped with a signal‐code for protein interaction and an altered intracellular localization, as well as degradation. All these modifications have been identified in tau aggregates obtained from diseased or transgenic brains (Cripps *et al*. 2006; Min *et al*. 2010; Funk *et al*. 2014) and, interestingly, they share a common set of lysine residues concentrated mostly around the microtubule‐binding domains (Morris *et al*. 2015), leading to the idea that there is a competitive cross‐talk between these modifications (Yang and Seto 2008).

Sumoylation is a process that tags proteins with small ubiquitin‐related modifiers, in a process similar to ubiquitination, but without ending in protein degradation. Sumoylation regulates various functional properties of many proteins, including tau. An *in vitro* study suggested that free tau is more accessible to sumoylation (Dorval and Fraser 2006). Although in Tg2576 mice small ubiquitin‐related modifiers and tau colocalize (Takahashi *et al*. 2008), tau lesions in affected human brains show low levels of sumoylated tau (Pountney *et al*. 2003), perhaps resulting from proteasome failure and increased tau ubiquitination (Dorval and Fraser 2006).

Oxidative stress can contribute to tauopathy as revealed by studies of pathological tau modifications caused by the formation of intermolecular bridges between cysteine residues (Schweers *et al*. 1995) or by nitration of tyrosines (Reyes *et al*. 2012). Moreover, reactive oxygen species (ROS) are highly reactive molecules that are intracellularly produced even under physiological conditions, by specialized organelles such as mitochondria, peroxisomes or the endoplasmic reticulum. Oxidative stress markers, together with ROS production, are acknowledged as signs of a possible evolution of tauopathy in both humans and animal models (Alavi Naini and Soussi‐Yanicostas 2015). Although tyrosine nitration has been observed in AD‐, CBD‐ and PiD‐affected brains (Horiguchi *et al*. 2003), the mechanisms that generate nitrated tau are still debated. In older studies, it was suggested that tau nitration is a random process resulting from the generation of free radicals during neurodegeneration (Ischiropoulos and Al‐Mehdi 1995), whereas more recently, tau nitration was suggested to be a highly regulated, lesion‐specific process (Reyes *et al*. 2012).

It is not entirely clear whether these alterations in tau structure represent an upstream event in tau pathology, whether tau is able to induce certain pathological cascades on its own, or whether a combination of both occurs. For example there might be a feedback loop by which pathological tau interferes with mitochondrial function and induces oxidative stress, which in turn pathologically alters tau structure (Alavi Naini and Soussi‐Yanicostas 2015).

## Cell cycle and epigenetics in tauopathies

Aberrant cell cycle activation in neurons has been reported in both tauopathies and animal models, providing a possible explanation for the generally late onset of neurodegenerative diseases (Herrup and Yang 2007). In aged mice expressing wild‐type 2N4R human tau in the absence of mouse tau, re‐expression of cell‐cycle regulatory proteins and DNA synthesis was induced, demonstrating that wild‐type tau pathology and neurodegeneration may be linked via abnormal, incomplete cell‐cycle re‐entry (Andorfer 2005). In a *Drosophila* tauopathy study, a neuroprotective role because of the induction of DNA damage checkpoint molecules has been suggested (Khurana *et al*. 2012). Based on serial analysis of gene expression of amygdala samples from pR5 mice, 29 differentially enriched transcripts were identified, including *Sfpq* that encodes a nuclear factor implicated in the splicing and regulation of gene expression (Ke *et al*. 2012). Furthermore, by analysing human AD and PiD brain samples, translocation of splicing factor proline/glutamine‐rich from the nucleus to the cytoplasm was observed in neurons and astrocytes, highlighting nucleo‐cytoplasmic redistribution of transcription factors as a pathomechanism in tauopathies (Ke *et al*. 2012). Tau‐related chromatin relaxation was observed in a study encompassing human R406W tau *Drosophila* and JNPL3 mouse models of tauopathy, as well as AD brain samples (Frost *et al*. 2014). Interestingly, in the *Drosophila* model, genetic manipulation of the genes related to heterochromatin reorganization resulted in a phenotypic rescue. These studies suggest a tauopathy‐related induction of gene expression of molecules implicated in neuronal dedifferentiation and re‐entry into a non‐appropriate cell cycle, correlated with chromatin restructuring and aberrant translocation of nuclear factors, which together leads to neurodegeneration and cellular loss.

## Role of tau in dendritic spines and synaptic impairment

Studies into how Aβ induces synaptic dysfunction have helped to understand how tau impairs synaptic function on its own. This is because how Aβ interacts with tau in causing synaptic dysfunction is, to some degree, understood although there are still major gaps to be filled (Tu *et al*. 2014). Aβ has been found to interact in its oligomeric and possibly fibrillar form with various synaptic and extra‐synaptic receptors, thereby triggering a series of toxic events. Specifically the activation of the N‐methyl‐D‐aspartate (NMDA) receptor, both directly and indirectly, together with increased calcium influx, has several downstream consequences, including hyperphosphorylation of tau and depolymerization of F‐actin, which ultimately results in synaptic dysfunction and cognitive impairment (Tu *et al*. 2014). Tau has a critical role in mediating the toxicity of Aβ in the synapse (Roberson *et al*. 2007; Vossel *et al*. 2010; Leroy *et al*. 2012). More specifically, it has been found that tau is required to target the Src kinase Fyn to the post‐synapse, where it phosphorylates NMDA receptor subunits which then recruit the adaptor protein PSD‐95 to facilitate the formation of an excitotoxic protein complex (Ittner *et al*. 2010). Furthermore, using hippocampal slice cultures, it was found that chemically inducing long‐term depression activated the NMDA receptor, together with reversible tau phosphorylation at several pathological epitopes (Mondragón‐Rodríguez *et al*. 2012).

Tau‐dependent synaptic dysfunction involves Fyn kinase (Ittner *et al*. 2010). This kinase contains an amino‐terminal Src‐homology (SH) region with acylation sites, a unique domain, an SH3 domain (with which it interacts with the PXXP motifs found in the proline‐rich region of tau) (Fig. 1b), an SH2 domain (with which it interacts with tau phosphorylated at residue Y18), an SH1/kinase domain and a carboxy‐terminal regulatory tail (Brandt *et al*. 1995; Lee *et al*. 1998). It has been shown that pseudophosphorylated tau binds Fyn more tightly than wild‐type tau, thereby increasing Fyn activity and synaptic damage (Bhaskar *et al*. 2005). Using real‐time surface plasmon resonance studies, it was further found that the interaction between the SH3 domain of Fyn and 3R‐tau was 20‐fold higher than that with 4R‐tau, and that the affinity between 4R‐tau and Fyn SH3 was 25–45‐fold increased in the presence of P301L tau (Bhaskar *et al*. 2005).

In V337M mutant mice, mutant tau depleted PSD‐95, which resulted in smaller post‐synaptic densities, impaired synaptic localization of NMDA receptors, and reduced firing of striatal neuron (Warmus *et al*. 2014). The NMDA receptor hypofunction contrasts with the hyperfunction and excitotoxicity caused by Aβ. In this context, a recent review discusses the possibility of sequential activation of Fyn, followed by its inactivating phosphatase, striatal‐enriched protein tyrosine phosphatase, in the regulation of the NMDA receptor, synaptic plasticity and the induction of synaptic depression, suggesting hyperfunction followed by hypofunction (Boehm 2013).

It is also important to highlight again that, although tau is conventionally perceived as an axonal protein, it is found in the dendrite, albeit at lower levels, and, to an even lesser extent, in dendritic spines (Ittner *et al*. 2010). The latter are present on pyramidal neurons where they represent the main post‐synaptic elements of cortical excitatory synapses. Spine localization is massively augmented by the presence of pathogenic tau mutations such as P301L, as well as when tau is pseudophosphorylated, whereas replacing critical phosphorylation sites with alanine abrogates spine localization (Hoover *et al*. 2010; Xia *et al*. 2015). When full‐length tau containing up to 20 phosphate residues (i.e. being more phosphorylated than tau in human FTLD‐tau brains) was generated in Sf9 cells and then purified, it readily formed oligomers, whereas fibrils were only rarely observed. Exposure of primary neurons to these tau preparations caused a reduction in spine density without affecting overall cell viability (Tepper *et al*. 2014).

Increased concentrations of phosphorylated tau produce a range of effects on synapses. These include the induction of synaptic dysfunction by impairing the surface expression of α‐amino‐3‐hydroxy‐5‐methyl‐4‐isoxazolepropionic acid receptors as well as a reduction in synaptic transmission (Hoover *et al*. 2010). Consistent with these findings, tau deletion or inhibition of tau hyperphosphorylation using a GSK‐3β inhibitor can prevent Aβ‐induced impairment of long‐term potentiation, a long‐lasting strengthening of the response of a post‐synaptic neuron to stimulation that occurs with repeated stimulation and has been linked to learning and long‐term memory (Shipton *et al*. 2011). The inducible Tg4510 mouse model expressing P301L tau was used to examine the effects of tau pathology on hippocampal glutamate regulation (Hunsberger *et al*. 2015). This study reported a 40% increase in hippocampal vesicular glutamate transporters which package glutamate into vesicles, and a 40% decrease in hippocampal glutamate transporter 1, the major transporter responsible for removing glutamate from the extracellular space. P301L tau expression resulted in an up to 7‐fold increase in potassium‐evoked glutamate release in the hippocampus and a significantly decreased glutamate clearance, correlated with memory impairments, suggesting a mechanism by which tau might mediate hyperexcitability (Hunsberger *et al*. 2015).

To clarify the relationship between tau aggregation and the loss of synapses and neurons, two lines of mice expressing human tau with or without P301L mutation were compared (Kimura *et al*. 2010). Interestingly, P301L tau transgenic mice exhibited neuronal loss and produced insoluble tau with advanced age but not synaptic loss or memory impairment. In contrast, wild‐type tau‐expressing mice neither exhibited neuronal loss nor produced insoluble tau but did exhibit synaptic loss and memory impairment. Moreover, P301L tau was less phosphorylated than wild‐type human tau, suggesting that tau phosphorylation is involved in synaptic loss, whereas tau aggregation is more involved in neuronal loss (Kimura *et al*. 2010).

Tau localization to spines has further been found to depend on neuronal activity, implying that tau serves a physiological function in spines, and that disease processes that impact on its spine localization will cause an impairment in synaptic function (Frandemiche *et al*. 2014). In the context of AD, exposing cortical cultures to synthetic preparations of Aβ oligomers was found to induce mislocalization of tau into spines under resting conditions, abrogating subsequent activity‐dependent synaptic tau translocation (Frandemiche *et al*. 2014). In another study, P301L mutant tau expression was targeted predominantly to layer II and III neurons of the entorhinal cortex, leading to ultrastructural synaptic alterations in hippocampal circuits, in the absence of robust cognitive deficits (Harris *et al*. 2012).

The role of Aβ in synapse loss is reasonably well established, but despite data linking tau to synaptic function, the latter's role in synapse loss remains largely undetermined. To address age‐dependent synaptic loss and dysfunction in P301L tau‐over‐expressing rTg4510 mice, multiphoton *in vivo* imaging was used to reveal an approximately 30% loss of apical dendritic spines in individual pyramidal neurons, suggesting selective vulnerability to tau‐induced degeneration. The data further indicated that the tau‐induced loss of a subset of synapses may be accompanied by compensatory increases in other synaptic subtypes, thereby preserving overall synapse density. Synaptosomal fractionation showed a significant decrease in the expression of several synaptic proteins, suggesting a functional deficit of the remaining synapses in the rTg4510 brain (Kopeikina *et al*. 2013). What dictates the underlying selective vulnerability is not understood.

Based on the reconstruction of almost 20 000 dendritic spines in fixed hippocampal tissue from AD patients by intracellular injections of Lucifer yellow and staining with specific anti‐tau phosphorylated antibodies, it was revealed that the diffuse accumulation of phosphorylated tau in a putative pre‐NFT state did not induce changes in the dendrites of pyramidal neurons, whereas the presence of tau aggregates forming intraneuronal NFTs was associated with progressive loss of dendritic spines and changes in their morphology (Merino‐Serrais *et al*. 2013). Although these findings differ from those of the P301L mouse study discussed above (Kimura *et al*. 2010), they have obvious implications for therapeutic interventions.

Connectivity changes induced by pathological tau have also been reported. The stratum lacunosum moleculare is a structure that serves as a connection hub between the entorhinal cortex and hippocampus, two brain regions that are particularly vulnerable in AD. A detailed structural study in P301L mutant mice revealed an impaired functional and structural organization of the entorhinal‐hippocampal complex, in particular of the synapses and myelinated axons in the stratum lacunosum moleculare, with the authors concluding that white matter pathology deserves further attention in tauopathy (Maurin *et al*. 2014). It is also known that sleep disturbances are frequent in AD. When 3xTg mice were subjected to sleep deprivation, there was a significant increase in the insoluble fraction of tau, and a reduction in PSD‐95 (Di *et al*. 2014). To the best of our knowledge, whether such changes are also found in pure tauopathy models has not been reported. Synaptic changes have not been extensively addressed in invertebrate models. A recent study in *Drosophila* expressing R406W tau identified 62 genes that, when silenced by RNAi, specifically modified tau‐induced toxicity. Among these were three subunits of the dynein/dynactin motor complex. In the course of a phenotypic analysis, owing to strong pathological changes in the axon, but not the synapse, the authors concluded that tau‐induced detrimental effects have an axonal rather than synaptic origin (Butzlaff *et al*. 2015).

Finally, the fact that homeostasis of tau levels is critical for neuronal functions is not only evident from FTLD‐tau cases, where an altered ratio of tau isoforms can cause neurodegeneration and cognitive decline, but also from the analysis of the *MAPT* locus for which microdeletions have been reported that may have a role in intellectual disability. Interestingly, by reducing tau levels in mice neuronal development or migration was inhibited (Caceres and Kosik 1990; Takei *et al*. 2000). In support, a more recent study observed that in the early postnatal brain, a number of neurons with reduced tau levels that reached the cortical plate exhibited underdeveloped dendrites and a striking reduction in connectivity as evidenced by the size of their boutons (Sapir *et al*. 2012). The authors concluded that *MAPT* may be a dosage‐sensitive gene involved in intellectual disability. Together, these findings indicate that tau has a role in the synapse and that the accumulation of hyperphosphorylated forms of tau, particularly in the spine, causes functional impairments and synaptic degeneration.

## Spreading of tau pathology

Tau aggregates appear to spread along neuronal pathways from one brain region to another. This aspect has been characterized extensively in AD, where tau accumulation is initiated in the locus coeruleus of the brainstem, before appearing in transentorhinal and entorhinal regions of the temporal lobe, followed by inclusions in the hippocampus and parts of the neocortex and, in the most severe cases, affecting almost the entire neocortex (reviewed in (Braak and Tredici 2011; Brettschneider *et al*. 2015). However, in FTLD‐tau, the aspect of this apparent tau spreading is less well characterized. A study focussing on the dynamics of tau deposition in PSP reported an initial pallido‐luyso‐nigral stage of tau deposition, progressing to a gradually increasing involvement of the basal ganglia, the pontine and dentate nuclei, and the frontal and parietal lobe (Williams *et al*. 2007). In AGD, on the other hand, the initial sites of deposition were found to be the ambient gyrus, the CA1 region of the hippocampus and the basolateral nucleus of the amygdala, then the entire amygdala, along with the involvement of the posterior transentorhinal cortex and the subiculum, and the anterior medial temporal lobe, whereas in the final stage of the disease, argyrophilic grains are also found in the insular cortex, the anterior cingulate gyrus, the nucleus accumbens, the septal nucleus, the hypothalamus and the gyri recti beyond the boundary of the temporal lobe (Saito *et al*. 2004). These data point towards a high level of heterogeneity in tau spreading. This difference in the pattern of spreading between AD and FTLD or even within FTLD‐tau may be explained by different triggers of the tau pathology. Moreover, it seems that AD requires abundant tau inclusions as well as Aβ deposits in the neocortex to trigger the disease clinically, whereas initial tau deposition appears to be insufficient for disease development (Jack *et al*. 2013; Duyckaerts *et al*. 2015). Overlapping the models of a gradual distribution with the connectivity maps of the brain led to the formulation of different spreading hypotheses, the most appealing of which is currently a ‘prion‐like model of propagation’ (Sanders *et al*. 2014; Goedert 2015). However, this model has been contested or at least modified in a recent study (Wegmann *et al*. 2015, commented further by Polanco and Götz 2015). Tau aggregation has been described as a heterogeneous nucleation reaction, whereby exogenous effectors, tau gene mutations or other modifications that stabilize assembly competent conformations of tau act to trigger the fibrillization reaction (Kuret *et al*. 2005). This concept has been strengthened by both *in vitro* and *in vivo* studies that used chemicals to induce tau phosphorylation and aggregation, such as in the case of okadaic acid mentioned earlier. In this regard, a recent study proposed the use of microfluidic chambers to study the spread of okadaic acid‐triggered tau phosphorylation in primary cultures containing healthy and diseased neurons (Kunze *et al*., 2011). However, the mechanisms leading to progressive tau phosphorylation remain elusive.

By injecting brain lysates from transgenic NFT‐forming P301S mice into pre‐symptomatic ALZ17 mice, filamentous and hyperphosphorylated tau species were detected at the injection site and in closely connected regions 6 months after injection, suggesting transmissibility of tau pathology (Clavaguera *et al*. 2009). This contributed to the application of the prion hypothesis to tau, in which pathologically altered tau protein strains recruit native tau, converting it into pathological species. To verify this hypothesis, human brain lysates from various human tauopathies were injected into ALZ17 brains, resulting in brain lesions, including NFTs, argyrophilic grains and Pick bodies using samples originating from AD, AGD and PiD cases respectively (Clavaguera *et al*. 2013). To understand which molecular species are responsible for the induction of tau phosphorylation, recombinant fibrils were injected into wild‐type mouse brains, revealing that only aggregated tau is capable of recruiting endogenous tau in a process mediated by extracellular heparin sulphate (Holmes *et al*. 2013). In another study, injection of recombinant tau fibrils into PS19 mice decreased the onset of NFT formation, suggesting that preformed synthetic fibrils are capable of inducing NFT‐like tau aggregates and initiating the spread of tau pathology (Iba *et al*. 2013). Intriguingly, increased tau phosphorylation was also detected after intraperitoneal application of lysates from transgenic P301S mice into heterozygous mice of the same line (Clavaguera *et al*. 2014).

However, it is not yet entirely understood what induces a self‐feeding mechanism of propagation, or whether there is another mechanism that responds to the injection of a mixture of not fully characterized foreign material into the brain. In support of this, two papers have highlighted a potential involvement of microglia in enhancing tau pathology and spreading of pathological tau in the brain, even though the mechanistic picture in not entirely clear. The first of these is based on the observation that mice lacking the microglial fractalkine receptor (Cx3cr1) crossed with those over‐expressing human 2N4R tau (Andorfer *et al*. 2003) present tau hyperphosphorylation and aggregation as early as 2 months of age, together with the spread of tau pathology to anatomically connected regions within the hippocampus (Maphis *et al*. 2015). Moreover, adoptive transfer of purified microglia from hTau/Cx3cr1^−/−^ mice induced tau hyperphosphorylation in non‐transgenic animals (Maphis *et al*. 2015). In another study, tau propagation was halted when microglia were depleted from the brain, suggesting their involvement in tau propagation supposedly mediated by exosome secretion (Asai *et al*. 2015). Recently, however, the prion model of tau spreading was contested or at least modified in a study that showed that pathological tau can propagate across neural systems in the absence of a templated misfolding and that the absence of endogenous tau greatly decreases its toxicity (Wegmann *et al*. 2015).

## Other tau‐related cellular dysfunctions

Steric hindrance of molecular function as well as possible organelle trapping because of sequestration of tau into the cytosolic pool have been hypothesized as factors that affect normal cellular function. In the 1970s, it was observed that tau contributes to microtubule stabilization (Weingarten *et al*. 1975), and it was shown subsequently that hyperphosphorylated tau sequesters normal tau into NFTs, thereby leading to microtubule disassembly (Alonso *et al*. 1996). Increased levels of unbound tau can also contribute to neuronal dysfunction even prior to the formation of detectable levels of tau deposits as shown in the Tg4510 mouse model (Santacruz *et al*. 2005).

It has been demonstrated that tau can interact with actin at a physiological pH, inducing actin filament bundles by interactions mediated by the microtubule‐binding domains (Moraga *et al*. 1993), or via its proline‐rich domain (He *et al*. 2009). An *in vitro* study revealed that over‐expression of different forms of tau can inhibit the plus‐end‐directed transport of vesicles along microtubules by kinesin such that the minus‐end‐directed transport by dynein becomes more dominant (Ebneth *et al*. 1998). As a consequence, exocytosis slows down and affects the distribution of mitochondria which become clustered near the microtubule‐organizing centre (Ebneth *et al*. 1998).

In *Drosophila*, R406W tau‐mediated actin stabilization inhibits the association of the fission protein dynamin‐related 1 with mitochondria, causing a shift in mitochondrial dynamics towards elongation and production of ROS, with some of these aspects being confirmed in tau transgenic mouse models (DuBoff *et al*. 2012). Pathological tau‐related mitochondrial dysfunction, including impaired oxidative phosphorylation, has been revealed by a proteomic and functional analysis of pR5 mice (David *et al*. 2005; Rhein *et al*. 2009). Interestingly, neuronal quality control of microglia is affected by a truncated form of human 2N4R tau (Amadoro *et al*. 2014). This study specifically revealed that early neuronal death correlated with dystrophic mitochondria dynamics, selective autophagic clearance, fragmentation and perinuclear mislocalization of small and dense mitochondria (Amadoro *et al*. 2014). Together, these studies highlight the critical role of mitochondrial turnover and function in FTLD pathology.

Alterations of normal actin dynamics will, however, also affect other cellular functions, such as the transport of membrane organelles via myosin‐dependent mechanics, an aspect that has been observed in models of tauopathies (Semenova *et al*. 2008). K369I mutant tau transgenic K3 mice are characterized by impaired axonal transport because of a tau‐dependent cargo‐selective impairment of kinesin‐driven axonal anterograde transport (Ittner *et al*. 2009). A more in‐depth analysis of these mice revealed that hyperphosphorylated tau interacts with c‐Jun N‐terminal kinase‐interacting protein 1 (JIP1), which becomes trapped in the soma and is not efficiently available to associate with the kinesin motor protein complex, thereby causing impaired axonal transport of defined cargoes (Ittner *et al*. 2009).

## Conclusions and perspectives

**Figure 3:**
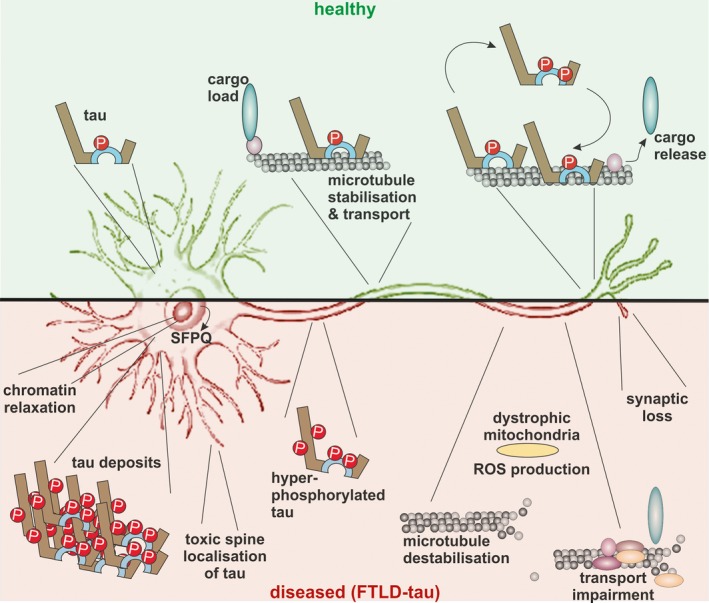
Tau‐mediated cellular functions under healthy and pathological conditions. In healthy neurons (top), tau molecules are found in a specific localization, with the highest concentrations being found within the distal part of the axon. Cargo molecules are particularly sensitive to the presence of a tau gradient within the axon. Disequilibrium of tau distribution (bottom), as found in frontotemporal lobar degeneration (FTLD)‐tau, has severe consequence for cellular physiology. Under these conditions, tau accumulates in the neuronal soma and dendrites, leading to microtubule depolymerization and affecting axonal transport. Mitochondrial impairments increase the production of toxic reactive oxygen species (ROS). Intra‐cytoplasmatic hyperphosphorylated tau aggregates are sequestrating more molecules, stalling cellular physiology. In the nucleus, the ratio between eu‐ and hetero‐chromatin is altered and transcription factors [e.g. splicing factor proline/glutamine‐rich (SFPQ)] can redistribute from the nucleus to the cytoplasm. Neuronal interconnectivity is lost as a result of a toxic accumulation of tau in dendritic spines, together with the loss of synaptic input.

Since the discovery of tau, many aspects of its physiology and pathology have been clarified, particularly with the development of improved *in vitro* and *in vivo* models (Fig. 3). Human neurons derived from embryonic stem cells or the more recently developed induced pluripotent stem cells (iPSCs) are now being explored to study tau‐associated pathomechanisms. In one study, the generation of neuroepithelial embryonic stem cells cultures was combined with lentiviral expression of 2N4R human tau or a pseudophosphorylated form of tau driven by the elongation factor 1α promoter (Mertens *et al*. 2013). Cells expressing the altered form of tau recapitulated the tau pathology seen in transgenic animals, together with degeneration and an increased rate of neuronal cell death. Moreover, these cellular studies confirmed the causative link between tau phosphorylation and conformation, microtubule‐based transport and the vulnerability of human neurons to oxidative stress (Mertens *et al*. 2013). In another study supporting prior findings in transgenic mouse models, it was revealed that seeding iPSCs with oligomeric, but not monomeric tau caused an increase in aggregated and pathologically phosphorylated tau, together with neurite retraction, loss of synapses, aberrant calcium homeostasis and an imbalance in neurotransmitter release (Usenovic *et al*. 2015). Recently, a 3D human cell culture system based on neuroprogenitor cells was used to model AD, successfully recapitulating both the extracellular aggregation of Aβ and an intracellular accumulation of hyperphosphorylated tau, opening the door to new technical possibilities for studying tauopathies *in vitro* (Kim *et al*. 2015).

Animal models have pointed towards the fact that tau levels need to be balanced. FTLD‐tau seems to be characterized by both a gain‐of‐toxic‐function and a loss‐of‐physiological function that together cause disease. However, it is becoming increasingly clear that, despite their merits, the existing models have also intrinsic limitations (Ittner *et al*. 2015). Based on the explosion of gene editing techniques, improved models of FTLD can be created, in the absence of the adverse effects inherent to protein over‐expression (Saito *et al*. 2014). Whether endogenous levels of expression of mutated genes will be generally sufficient to achieve pathology within the lifespan of rodents remains to be determined. Nevertheless, deciphering the molecular pathogenesis of FTLD offers new perspectives for the development of therapies, which is critical to prevent or halt tauopathies. As novel interventions are being developed, the animal models will be instrumental in validating treatment options.
